# The Relationship between Therapeutic Alliance and Service User Satisfaction in Mental Health Inpatient Wards and Crisis House Alternatives: A Cross-Sectional Study

**DOI:** 10.1371/journal.pone.0100153

**Published:** 2014-07-10

**Authors:** Angela Sweeney, Sarah Fahmy, Fiona Nolan, Nicola Morant, Zoe Fox, Brynmor Lloyd-Evans, David Osborn, Emma Burgess, Helen Gilburt, Rosemarie McCabe, Mike Slade, Sonia Johnson

**Affiliations:** 1 Division of Psychiatry, UCL, London, United Kingdom; 2 Centre for Outcomes Research and Effectiveness (CORE), UCL, London, United Kingdom; 3 University of Cambridge, Cambridge, UK & UCL, London, United Kingdom; 4 Joint Research Office and Institute of Neurology, UCL, London, United Kingdom; 5 Institute of Psychiatry, Kings College London, London, United Kingdom; 6 University of Exeter Medical School, Exeter, United Kingdom; 7 Ment Division of Psychiatry, UCL, London, United Kingdom; 8 Camden and Islington NHS Foundation Trust, London, United Kingdom; University of Michigan, United States of America

## Abstract

**Background:**

Poor service user experiences are often reported on mental health inpatient wards. Crisis houses are an alternative, but evidence is limited. This paper investigates therapeutic alliances in acute wards and crisis houses, exploring how far stronger therapeutic alliance may underlie greater client satisfaction in crisis houses.

**Methods and Findings:**

Mixed methods were used. In the quantitative component, 108 crisis house and 247 acute ward service users responded to measures of satisfaction, therapeutic relationships, informal peer support, recovery and negative events experienced during the admission. Linear regressions were conducted to estimate the association between service setting and measures, and to model the factors associated with satisfaction. Qualitative interviews exploring therapeutic alliances were conducted with service users and staff in each setting and analysed thematically.

**Results:**

We found that therapeutic alliances, service user satisfaction and informal peer support were greater in crisis houses than on acute wards, whilst self-rated recovery and numbers of negative events were lower. Adjusted multivariable analyses suggest that therapeutic relationships, informal peer support and negative experiences related to staff may be important factors in accounting for greater satisfaction in crisis houses. Qualitative results suggest factors that influence therapeutic alliances include service user perceptions of basic human qualities such as kindness and empathy in staff and, at service level, the extent of loss of liberty and autonomy.

**Conclusions and Implications:**

We found that service users experience better therapeutic relationships and higher satisfaction in crisis houses compared to acute wards, although we cannot exclude the possibility that differences in service user characteristics contribute to this. This finding provides some support for the expansion of crisis house provision. Further research is needed to investigate why acute ward service users experience a lack of compassion and humanity from ward staff and how this could be changed.

## Introduction

Inpatient care is a key component of mental health systems across higher and middle income societies. However, the evidence base regarding acute care outcomes is weak and there is not yet a clear consensus about the aims of admissions (beyond risk management), the content of care, or what elements make inpatient stays effective [1], [2]. Qualitative and survey research studies in mental health in-patient settings have identified a number of concerns. Most notably, many service users are dissatisfied, describing wards as non-therapeutic and frightening [3], [4], [5]. Poor relationships between staff and service users are frequently reported [6], [7], [8]. Inpatient ward staff identify several barriers to developing therapeutic relationships including: low staffing levels; an associated lack of staff continuity; bureaucratic demands; and uncertainty about the adoption and implementation of therapeutic models [9]. Unsurprisingly, reforming acute care was recently identified as the highest priority of mental health staff and service users in England [10].

Residential crisis alternatives have been developed as one solution to the problems posed by inpatient care. Crisis houses tend to be smaller than their hospital counterparts, with a more domestic atmosphere [9], [11]. They are generally well-embedded in the local healthcare system, serving similar populations to inpatient wards, although they rarely admit people compulsorily and fewer people have a history of violence [9], [11]. Force, restraint and seclusion are rarely used. Despite a long history (which dates back at least to the 1960s), there is little robust research examining crisis houses. However, a recent systematic review of predominantly American literature found that service user satisfaction is greater in crisis residential alternatives than on inpatient wards [12] with some research evidence that the quality of staff-service user relationships is enhanced [13].

Several of the authors were involved in a precursor to this study, the Alternatives Study, which employed mixed research methods to investigate and compare inpatient crisis care with residential alternatives [9], [11], [14]–[19]. Service user populations were in many respects found to be similar in hospitals and in crisis houses, for example with no significant differences in employment rates, previous history of psychiatric hospital admission and recent history of self harm (11). Crisis house residents were more likely to be already known to secondary mental health services prior to admission than those admitted to hospital, and they were also less likely to have a history of violence. They were more likely to have initiated help-seeking themselves in the current crisis: however, for 69% of crisis house admissions (compared with 83% of ward admissions), either health staff or a family member had initially sought help. Our quantitative findings confirmed considerably greater service user satisfaction with crisis houses than with inpatient wards (17). However, potential explanatory variables (such as the amount of contact between staff and service users, the types of intervention provided and service outcomes) did not differ significantly between the two settings. Whilst we did not assess therapeutic alliances quantitatively, our qualitative findings suggested that these might be key to service users' satisfaction with crisis care. Staff participants felt that characteristics of crisis houses such as the home-like environment and promotion of autonomy created greater opportunities for developing strong therapeutic relationships than in hospital [20]. Relationships with peers, coercion, safety, and the extent of exposure to other service users who were aggressive and disturbed also emerged as potentially important influences on satisfaction [16].

The aim of the current study is to explore these factors and their relationship with service user satisfaction. A mixed methods cross-sectional design has been used to explore therapeutic alliance and its relationship to service user satisfaction in community residential and standard inpatient services. The specific objectives of the paper are:

1. To test the primary hypothesis that therapeutic alliance between staff and service users is stronger in residential crisis alternatives than in standard inpatient settings.
2. If hypothesis one is confirmed, to examine how far, adjusting for other potential explanatory variables, better therapeutic alliances may account for greater satisfaction in crisis houses.
3. To explore the association with satisfaction of a number of other potential explanatory factors identified in the Alternatives Study, namely self-rated recovery, relationships with staff, informal peer support and experiences of negative events.
4. To use qualitative methods to develop understanding of the factors that influence therapeutic alliance from service user and staff perspectives.

As is increasingly common in health services research, the study was conducted by a multidisciplinary team including researchers with clinical backgrounds in psychiatry, psychology, nursing and social work, three service user researchers, two qualitative experts and a statistician.

## Methods

### Study design and setting

The quantitative component of the study used structured interviews for service users in a cross-sectional design to test our primary hypotheses and to generate a model of service user satisfaction. The qualitative component used semi-structured interviews with staff and service users in the same settings to explore their perspectives on and experiences of therapeutic alliances, with a particular focus on the barriers and facilitators to positive therapeutic relationships.

The study was conducted in 16 inpatient wards located in two neighbouring National Health Service (NHS) Trust catchment areas in inner London, United Kingdom, and four crisis houses in the same catchment areas. The catchment areas are inner city areas with high levels of ethnic diversity and social deprivation. The crisis houses vary from services within the statutory sector staffed predominantly by qualified mental health clinicians to services within the voluntary sector predominantly employing social care staff. One crisis house is only for women. All the crisis houses are closely linked into the local catchment area acute service systems, with crisis resolution teams the primary referrers to both wards and crisis houses. Who goes where in a crisis is typically determined by a combination of staff decision making, service user preferences and where beds are available. Quantitative data were collected in all 20 services, and qualitative data in all four crisis houses and five wards. Data were collected between January 2011 and November 2012.

## Quantitative component of the study

### Samples and procedures

We recruited service users with a good level of English who were able to provide informed consent and who had been resident in a crisis house for a minimum of five days (one site with a short average stay) or seven days (remaining sites), or resident on a ward for at least two weeks. Service users were sampled consecutively where possible, and included in the study where they met our inclusion criteria and consented to participate. Written informed consent was sought prior to interview. Participants provided basic socio-demographic and clinical information and completed a number of measures, described below.

The data from inpatient wards were collected primarily for a sister study, the Protected Engagement Time Study (PET) (http://public.ukcrn.org.uk/Search/StudyDetail.aspx?StudyID=7802). This study was an evaluation of an intervention designed to increase the quantity and quality of staff and service user interaction on inpatient wards. Our study was designed to make further use of the PET study data, so that inclusion criteria, data collection tools and procedures used in the crisis houses for the current study matched those used in the wards for the PET study. Participants in the PET study gave written informed consent for data to be shared between the two studies.

We calculated that a sample of 85 service users per arm (wards versus crisis houses) would provide 90% power to detect a medium standardised effect size in the average STAR-P rating of 0.5 at the 5% significance level. Inflation for the clustered nature of the data required a final sample of 108 per arm.

### Measures

The following measures were completed by service users in interviews with a study researcher.

Socio-demographic and clinical data were collected, including gender, age, ethnic group, country of birth, mental health diagnosis as recorded in clinical records, Mental Health Act status and admissions history. These data were confirmed, where possible, from clinical records.

The **Scale to Assess Therapeutic Relationships – Patient version** (STAR-P) is a 12-item measure assessing the relationship between service users and staff on three components: collaboration, positive clinician input, and non-supportive clinician input [21]. Participants rated their alliance for their keyworker or the person they had worked with the most and two additional staff members whom they considered important in their care. The STAR-P score used in the main analysis was the mean of all STAR-P ratings completed by each service user. Where only two staff members were rated, the mean of these was taken. The range of scores is 0–48, with a higher score suggesting better therapeutic relationships. As a secondary measure, participants rated their relationship with the staff group at the service as a whole. This is shown in the analyses as ‘general staff STAR’.

The **Client Satisfaction Questionnaire** (CSQ) [22] is an 8-item measure assessing service user satisfaction with services. The range of scores is 8 to 32, with a higher score suggesting greater satisfaction and a score of 20 indicating a neutral - neither satisfied nor dissatisfied - perspective.

An abbreviated version of the **Interpersonal Relationship Inventory** (IPR) [23] was used to measure informal peer support. The IPR measures informal social support in a range of health settings. To assess relationships with other service users we selected the support and conflict subscales which together contain 26 items. This generates a total score between 0 and 104, with a higher score indicating better interpersonal relationships.

The **Recovery Assessment Scale** (RAS) [24] is a 41-item measure of recovery–related concepts such as hope, empowerment and connection. The RAS generates a total score ranging from 0 to 164, with a higher score indicating greater recovery.

To assess negative events experienced on the ward and in the crisis house we developed a **Negative Events Schedule for staff** (NES-S) and **for patients** (NES-P). These measures were developed for use in this study and the PET study because we were unable to identify any existing measures of negative events. The NES-S measures negative events perpetrated by staff and experienced during the current admission (e.g. being ignored by a staff member, being sexually assaulted by a staff member) whilst the NES-P measures negative events perpetrated by other service users during the current admission (e.g. being ignored by another service user, being sexually assaulted by another service user). Items were derived for the NES initially from a measure developed for a previous study [9] and discussed with a working group of service users and staff and a service user research reference group in a series of meetings. The final schedules were piloted with service users to assess feasibility and acceptability [25]. Further psychometric properties have not been tested. In the final schedules, participants are asked whether the item/event has been experienced (yes/no), the approximate number of times, and the level of impact (0–4 Likert scale from ‘none’ to ‘a great deal’). Weighting for impact was introduced at the suggestion of service users who felt this was important. To calculate a total negative event score, negative events with no impact were scored 0 and negative events with a great deal of impact were scored 4 (maximum impact). Scores were then summed separately for NES-S (total score range 0–56) and NES-P (total score range 0–48).

### Data management and analysis

Following data checking and cleaning, we constructed tables of descriptive statistics for the sample. Linear regression with therapeutic relationship (STAR-P, using the mean of three staff questionnaires) as the outcome measure and service setting (crisis house versus ward) as the sole explanatory variable was then carried out as an initial test of the primary hypothesis (that therapeutic alliances are stronger in the crisis house). Adjustment was made for clustering within the data, clusters being all the service users admitted to a particular crisis house or ward. The estimate of the association between service setting and therapeutic relationships was adjusted for the following potential confounders: age, ethnic group, sex, length of stay prior to the study interview, history of previous admission, whether detained under the Mental Health Act during this admission, and diagnosis.

Subsequent main steps in the analysis involved exploring variables associated with service satisfaction. Initially mean score for service satisfaction (CSQ) was compared between crisis houses and hospital, adjusted for the demographic, diagnostic and service use variables listed above. We then further added to this model as explanatory variables therapeutic relationship (STAR-P), relationship with peers (IPR), extent of recovery (RAS), and negative events (NES-S, NES-P). This resulted in a final multiply adjusted model of the explanatory variables associated with satisfaction.

## Qualitative component of the study

### Samples and procedures

The inclusion criteria and procedures were the same as in the quantitative component except that all participants were resident in crisis houses for at least one week. Furthermore, we used purposive sampling to ensure that we interviewed staff from a range of professional and socio-demographic backgrounds and service users who were similar in socio-demographic characteristics and admission histories to the whole population of users of the services in the preceding 12 months. The majority of service user interviews (93%) were conducted by service user researchers.

### Interview schedules

Interview schedules were developed to explore therapeutic alliances between staff and service users in acute settings, with a particular focus on understanding the facilitators and barriers to positive therapeutic alliances. Schedules were grounded in previous research findings [16], [20], piloted with staff and service users and finalised in collaboration with service user researchers and other study team members. The final interview schedules focussed on expectations, characteristics, preferences, barriers, facilitators and recommendations surrounding therapeutic alliances. Service users and staff additionally considered a range of factors deemed potentially relevant for therapeutic relationships for each group, such as atmosphere, the personal qualities of staff, management and supervision. Participants with relevant experience were asked to compare their experiences of therapeutic alliances in different acute care settings.

### Data analysis

The analysis aimed to explore the factors that hinder and enhance therapeutic alliances. Thematic analysis [26] within NVivo software was used, and a collaborative approach involving several members of the research team was adopted. A service user researcher generated an initial coding frame following a detailed reading of transcripts. This was applied to the data to test its feasibility and fit. Study group members then attended a multiple coding meeting where the coding frame and higher level themes were discussed in depth. Research team members read a selection of transcripts and then met to discuss the appropriateness of the initial coding frame, competing explanations of the data and higher order emergent themes. Discussions were used to revise the coding frame and raise the level of abstraction. Data were then coded by two researchers who met frequently to enhance the consistency and reliability of coding. During this process the coding frame continued to evolve in line with emerging findings.

### Ethics Statement

Ethics approvals were gained from North West London Research Ethics Committee 1 (Reference 10/H0722/88). Approvals for the PET study were granted by the North East London Research Ethics Committee (Reference 09/HO711/87), including approval for the additional use of the data in the present study.

## Results

### Quantitative results

#### Sample characteristics

355 service users participated in the study, 108 in the crisis house group (85% of all those eligible) and 247 in the inpatient ward group (72% of those eligible). Participant characteristics can be found in Table 1 and scores on the main measures for the crisis house group and the inpatient ward group in Table 2. Crisis houses admitted more women (partly reflecting the fact that one crisis house admitted women only); more service users from a white British background; people who had been admitted more often to hospital or a crisis house in the past; just one person detained compulsorily (compared with two thirds of the inpatient ward group); fewer people diagnosed with psychosis and more people diagnosed with depression or personality disorder.

**Table 1:** Quantitative participant characteristics.

Characteristic		Crisis houses N = 108	Acute wards N = 247
**Gender**	male n (%)	38 (35%)	141 (57%)
**Age**	mean years (SD)	41 (13)	40 (13)
**Ethnic group**	n (%)		
	White British	63 (59%)	75 (30%)
	White Other	17 (16%)	24 (10%)
	Black Caribbean or African	9 (8%)	70 (28%)
	Asian	4 (4%)	34 (14%)
	Mixed Heritage	12 (11%)	12 (5%)
	Other	2 (2%)	32 (13%)
**Time in service centre prior to interview**	median weeks (IQR)	1.3 (1.0, 1.9)	5.6 (3.0, 10.0)
**Lifetime admissions to psychiatric hospital**	n (%)		
	0	24 (23%)	54 (22%)
	1	11 (10%)	44 (18%)
	2–5	33 (31%)	105 (43%)
	6–10	21 (20%)	33 (13%)
	>10	16 (15%)	11 (4%)
**Mental Health Act status at admission**	n (%)	1 (1%)	165 (67%)
**Current/most recent clinical diagnosis from clinical records**	n (%)		
	Schizophrenia/schizo-affective	18 (18%)	120 (56%)
	Bipolar disorder	17 (16%)	36 (17%)
	Other psychosis	3 (3%)	4 (2%)
	Depression	31 (30%)	11 (5%)
	Personality disorder	25 (24%)	16 (8%)
	Other	9 (9%)	26 (12%)

**Table 2:** Satisfaction, therapeutic alliance and other measures of patient experience in crisis houses and acute wards.

Measure		Crisis houses N = 108	Acute wards N = 247
**Satisfaction: CSQ total**	mean (95% CI)	27.5 (26.6, 28.3)	21.0 (20.2, 21.8)
**Therapeutic alliance: Average STAR**	mean (95% CI)	37.2 (35.5, 38.8)	28.3 (27.1, 29.5)
**Therapeutic alliance: General STAR**	mean (95% CI)	36.5 (34.7, 38.2)	25.6 (24.2, 27.0)
**Recovery: RAS total**	mean (95% CI)	102.3 (97.2, 107.4)	120.9 (117.1, 124.8)
**Informal peer support: Adapted IPR total**	mean (95% CI)	68.4 (65.5, 71.3)	57.1 (54.9, 59.3)
**Negative events committed by service users weighted by impact: NES-P**	median (IQR)	0 (0, 3)	3 (1, 8)
**Negative events committed by staff weighted by impact: NES-S**	median (IQR)	0 (0, 2)	3 (0, 9)

#### The relationship between service setting and therapeutic alliance

There was a large and highly significant difference in ratings of therapeutic alliance between crisis houses and inpatient wards, with the mean STAR-P score of inpatient ward participants −8.74 points lower (95% CI: −12.3, −5.19) than crisis house participants (p<0.0001, see Table 3). Age and gender showed statistically significant associations with therapeutic alliance (each increase of 5 years in participant's age was associated with a 0.4 [95% CI: 0.05, 0.15] increase in STAR-P score, whilst being female was associated with a reduction in STAR-P score of 1.73 [95% CI: −3.74, 0.29], see Table S1).

**Table 3:** Mean differences between crisis houses and inpatient wards on key measures of service user experience.

Dependent variable^1^	Regression Coefficient^2^	95% Confidence Interval	P
**Therapeutic alliance: STAR-P**	−8.74	−12.30, −5.19	<0.0001
**Satisfaction: CSQ**	−5.26	−7.59, −2.94	<0.0001
**Recovery: RAS**	2.36	−4.90, 9.62	0.51
**Informal peer support: IPR**	−12.08	−18.53, −5.63	<0.001

1 Details for scoring each measure can be found in the ‘measures’ sub-section of the methods section.

2 Adjusted for age, gender, ethnicity, diagnosis, time in service centre prior to interview, whether admitted to a psychiatric hospital in the past and Mental Health Act status at admission.

#### The relationship between service setting and other aspects of service users' experiences

There was a large and highly significant difference in satisfaction ratings between crisis houses and hospital (Table 3). Crisis house participants' average scores fell between satisfied and very satisfied (the mean score was 27.5, with 32 being the maximum possible score) whilst inpatient ward participants' scores were slightly better than neutral (the mean score was 21, with a score of 20 being neutral) with the difference significant at the p<0.0001 level (95% CI: −7.59, −2.94). None of the control variables was significant in the multivariable analysis (see Table S2).

A significant difference was found between the stage of recovery that participants in each of the groups rated themselves as having currently reached, with service users on wards more likely to rate themselves as at a relatively advanced stage of recovery than those in the crisis houses (the difference between mean scores in each setting was 18.59 (95% CI: 11.56, 25.63) and this was significant at p<0.0001). However, no baseline measurement had been made, and when adjustment was made for demographics, diagnoses and service use variables, this difference was no longer statistically significant (the mean difference fell from 18.59 to 2.36, 95% CI: −4.90, 9.62, Table 3). This appeared to be because diagnosis was strongly associated with self-rated recovery: people diagnosed with personality disorder had a 31.41 (95% CI: −41.36, −21.47) lower mean adjusted score than those diagnosed with schizophrenia or schizoaffective disorder, and people diagnosed with depression had a 20.72 (95% CI: −31.91, −9.53) lower mean adjusted score than those diagnosed with schizophrenia or schizoaffective disorder (see Table S3,).

A highly significant difference was found between ratings of informal peer support in each setting, with those in crisis houses rating this 12.08 points higher than those on inpatient wards (p<0.0001, see Table 3 and Table S4).

Finally, participants reported whether they had experienced negative events relating to other service users and to staff, and the impact of these (see Table 4). Adverse events relating to both groups were more frequent on inpatient wards for every negative event we asked about (with the exception of being offered illicit substances or alcohol by staff which had not been experienced by any participant).

**Table 4:** Negative events reported to have been perpetrated by staff (NES-S) and by service users (NES-P).

Negative events perpetrated by service users (NES-P)			
Characteristic	Crisis House N = 108 n yes (%)	Acute Ward N = 247 n yes (%)	P-value
Theft of personal belongings	4 (4%)	79 (32%)	<0.0001
Offered Illicit substances or alcohol	7 (7%)	30 (12%)	0.12
Verbal threats	6 (6%)	65 (26%)	<0.0001
Verbally abused	11 (10%)	70 (28%)	<0.0001
Physically assaulted	1 (1%)	30 (12%)	<0.0001
Sexually harassed	1 (1%)	21 (9%)	0.007
Sexual assaulted	0 (0%)	5 (2%)	0.33
Victim of religious, racial or homophobic discrimination	6 (6%)	33 (13%)	0.03
Forced to do something	4 (4%)	21 (8%)	0.11
Dismissed or ignored	24 (23%)	52 (21%)	0.71
Witnessed disturbed behaviour	37 (35%)	182 (74%)	<0.0001
Other	3 (3%)	8 (3%)	0.84

Negative events perpetrated by staff (NES-S)			
Characteristic	Crisis House N = 108 n yes (%)	Acute Ward N = 247 n yes (%)	P-value
Theft of personal belongings	0 (0%)	25 (10%)	<0.0001
Offered Illicit substances or alcohol	0 (0%)	0 (0%)	-
Verbal threats	2 (2%)	23 (9%)	0.01
Verbally abused	2 (2%)	24 (10%)	0.01
Physically assaulted	0 (0%)	13 (5%)	0.01
Sexually harassed	0 (0%)	6 (2%)	0.18
Sexual assaulted	0 (0%)	2 (1%)	0.99
Victim of religious, racial or homophobic discrimination	3 (3%)	42 (17%)	<0.0001
Forced to do something	5 (5%)	46 (19%)	0.001
Dismissed or ignored	18 (17%)	101 (41%)	<0.0001
Physically restrained	2 (2%)	57 (23%)	<0.0001
Spent time in quiet room	4 (4%)	54 (22%)	<0.0001
Given medication against will	2 (2%)	94 (38%)	<0.0001
Other	18 (17%)	11 (4%)	<0.0001

#### Modelling of factors associated with service user satisfaction

Table 5 shows the results of modelling the factors associated with service user satisfaction. Recovery was not associated with satisfaction on initial testing and so was excluded from the multivariable model. In the linear regressions between satisfaction and the main outcome measures (therapeutic alliance, self-rated recovery and informal peer support – note that negative events are not included at this stage as NES-S and NES-P do not have established psychometric properties), therapeutic alliance and informal peer support were significantly related to satisfaction (a 10 point increase in STAR score was associated with a 2.49 [95% CI: 1.51, 3.48;] increase in CSQ score (p<0.0001), whilst a 10 point increase in informal peer support score was associated with a CSQ increase of 0.74 [95% CI: 0.16, 1.31; p<0.01]). When these variables were included in an adjusted model of satisfaction and setting, the mean difference in satisfaction scores between crisis houses and inpatient wards fell from 5.26 to 2.44 (95% CI: −4.76, −0.13; p = 0.04). These results are compatible with the idea that better therapeutic alliance and informal peer support may be important factors in accounting for service users' increased satisfaction with crisis houses.

**Table 5:** Linear regression investigating how far therapeutic alliance, peer support and negative events are associated with satisfaction.

		Multivariable model excluding negative events		Multivariable model including negative events	
Characteristic		Coefficient (95% CI)	P-value	Coefficient (95% CI)	P-value
**Service type**	ward versus crisis house	−2.44 (−4.76, −0.13)	0.04	−1.38 (−3.16, 0.4)	0.12
**Gender**	female versus male	−0.22 (−1.37, 0.93)	0.70	0.54 (−0.51, 1.6)	0.3
**Age**	per 5 years older	0.03 (−0.17, 0.23)	0.76	−0.02 (−0.24, 0.21)	0.87
**Ethnic group**	White British	Reference group	0.59	Reference group	0.56
	White Other	−0.29 (−1.93, 1.36)		−0.14 (−1.68, 1.4)	
	Black	−0.91 (−2.96, 1.14)		−1.19 (−3.03, 0.65)	
	Asian	−0.39 (−2.10, 1.33)		−0.6 (−2.45, 1.25)	
	Mixed heritage	−0.35 (−2.06, 1.37)		−0.15 (−2.15, 1.84)	
	Other	−2.21 (−5.30, 0.87)		−1.01 (−3.9, 1.88)	
**Time in service centre prior to the interview**	per week longer	−0.00 (−0.08, 0.08)	0.94	−0.00 (−0.07, 0.06)	0.97
**Therapeutic alliance: Average STAR score**	per 10 unit higher	2.49 (1.51, 3.48)	<0.0001	2.19 (1.39, 2.98)	<0.0001
**Informal peer support: IPR total score**	per 10 unit higher	0.74 (0.16, 1.31)	0.01	0.5 (0.08, 0.92)	0.02
**Admitted to psychiatric hospital in the past**	yes versus no	−0.02 (−1.28, 1.24)	0.97	0.28 (−0.84, 1.4)	0.61
**Mental Health Act status at admission**	detained versus not detained	−1.32 (−2.81, 0.17)	0.08	−1.26 (−2.72, 0.19)	0.09
**Current/most recent clinical diagnosis**	Schizophrenia/schizo-affective	Reference group	0.05	Reference group	0.15
	Bipolar disorder	−1.54 (−2.95, −0.13)		−1.12 (−2.73, 0.5)	
	Other psychosis	2.75 (−2.42, 7.91)		2.23 (−2.14, 6.61)	
	Depression	−0.91 (−2.70, 0.89)		−1.16 (−2.93, 0.62)	
	Personality disorder	−1.29 (−3.70, 1.12)		−1.44 (−3.77, 0.88)	
	Other	−0.87 (−2.83, 1.10)		−1.3 (−2.97, 0.37)	

When negative events were added to the model, the relationship between staff-related negative events and client satisfaction was highly significant, with a fall in CSQ score of 0.35 (95% CI: (−0.52, −0.18) per additional weighted event. Service user-related negative events were not significantly related to satisfaction. In the resulting model, the difference between ward and crisis houses in satisfaction fell to 1.38 and was no longer statistically significant. This is compatible with the idea that an explanatory model involving therapeutic relationships, informal peer support and negative experiences related to staff may account for the greater satisfaction found in crisis houses than in wards.

### Qualitative participant characteristics

A summary of the characteristics of qualitative participants can be found in Table 6. Twenty nine service users were recruited, 14 from crisis houses and 15 from inpatient wards. The characteristics of service user participants were broadly in line with our purposive sampling targets, although we had anticipated recruiting more crisis house participants with experiences of psychosis.

**Table 6:** Characteristics of qualitative participants.

		Crisis house service users N = 14	Acute ward service users N = 15	Crisis house staff N = 6	Acute ward staff N = 7
Characteristics		N	N	N	N
**Gender**	Female	8	7	5	4
	Male	6	8	1	3
**Age**	Under25	2	2	0	0
	25–55	10	12	5	6
	Over55	2	1	1	1
**Ethnic group**	WhiteBritish	9	6	3	0
	WhiteOther	1	1	2	0
	BlackCaribbeanorBlackAfrican	3	3	1	5
	Asian	1	2	0	1
	Mixedheritage	1	2	0	1
	0	5	4	-	-
**Number of Previous Hospital Admissions**	1	2	1	-	-
	2–5	4	5	-	-
	6+	3	5	-	-
**Diagnosis**	Psychosis	5	12	-	-
	Non-psychosis	7	3	-	-
	Missing	2	0	-	-
**Had previously worked in the other service type**	Yes	-	-	3	2
**Role**	Manager/ClinicalPracticeLead	-	-	1	2
	Nurse	-	-	0	3
	SeniorProjectWorker	-	-	2	-
	ProjectWorker	-	-	3	-
	Other			0	2
**Professional background**	Qualifiednurse/counsellor/othermentalhealthprofessional	-	-	2	6
	Noclinicalqualifications	-	-	3	1
**Years in current service type**	<1	-	-	2	0
	0–5	-	-	3	3
	5+	-	-	1	4

Thirteen staff members were recruited, six from crisis houses and seven from inpatient wards. The majority of crisis house staff were female and from a white British or white other background. All inpatient ward staff were from a Black or other non-White background, with an approximate balance of men and women. We believe that this sample is broadly representative of the staff groups in the services, though with white groups slightly under-represented in the ward sample.

### Key thematic findings relevant to understanding differences between settings in relationships and experiences

In this paper we focus on aspects of the qualitative data that can contribute to understanding the reasons for the differences between inpatient wards and crisis houses identified in the quantitative component of the study. PP refers to the participant and IV the interviewer.

### Individual staff qualities

#### Basic human qualities lie at the heart of all therapeutic relationships

Underpinning positive therapeutic alliances are the basic human qualities of staff and their ability to communicate these to service users. All service users valued relationships with staff who demonstrated kindness; warmth; empathy; honesty; trustworthiness; reassurance; friendliness; helpfulness; calmness; and humour.

*PP It's sense of humour, calmness, ah, inner serenity, um…*

*IV So it's really about the personal qualities of staff?*

*PP I think it's more the personal quality, rather than what qualifications they've got, exactly, rather than what qualifications they've got. Definitely, definitely, yes.*

(Crisis house service user).

Similarly, staff in both crisis houses and on inpatient wards, described the basic human qualities that they believed underpinned their working style including warmth; humour; empathy; respect (e.g. of privacy, beliefs and preferences); honesty; fairness; and communication and listening. The ability to inspire hope and to understand service users as whole, unique individuals were also seen as important.

Service users often felt unable to build relationships with staff who did not seem to them to possess these basic human qualities and who, as a consequence, were seen as wrong for the job. Inappropriate personal qualities included: a lack of care and compassion; rudeness; disrespectfulness; untrustworthiness; insincerity; and a confrontational or belittling interactional style.

#### A vocation, not just a job

Service users in both settings described forming stronger therapeutic relationships with staff who were seen as dedicated to their profession. Such staff members were often described as dependable; compassionate; reliable; knowledgeable; and as going the extra mile. Service users also valued relationships with staff who were professional and observed clear boundaries, but who at times stepped beyond their professional role to respond to individual situations with warmth and humanity.

Conversely, where staff were seen as simply doing a job - that is, were viewed as uninterested in and disconnected from their work, and as simply being present to collect a wage - therapeutic alliance was hindered. It was more frequent for staff on inpatient wards to be viewed in this way, and although service users typically avoided these staff members, they could still exert a negative influence:

*Sometimes, ah, certain members of staff, they can be a bit difficult, and then with… because of their attitude; they don't have the right attitude for the job, really. They're supposed to be a carer, and the guy doesn't care. He just cares about himself; he doesn't care about the people, he just wants to get his money and go home. He wants to keep his job, basically, but makes your life hell while he's doing it.*

(Inpatient ward service user).

Some crisis house staff described seeking an appropriate balance between maintaining professional boundaries and responding to an individual with warmth and humanity. Achieving this was thought to require reflexivity and self-awareness. Whilst similar issues were sometimes raised by inpatient ward staff, they tended to be described in different ways (e.g. “*I'm firm but I'm fair*”). A small number of inpatient ward staff described witnessing poor practice by colleagues, including lacking empathy and patience, ignoring service users and not sharing workloads fairly. One person described this as a negative aspect of ward culture.

*a lot of staff here have probably got to that stage where they've been here too long and they're just doing the job just to have a job rather than doing the job because they enjoy the relationships they have with the patients and the actual what the job entails, patient care.*

(Inpatient ward staff member).

These descriptions of poor practice were strikingly similar to service users' accounts of poor therapeutic alliances.

#### Levels of interest and engagement

Service users formed better therapeutic alliances with staff members who appeared engaged with and interested in them. Even a simple act, such as asking someone how their day was going, could have important implications.

*IV When they're being really nice, what kind of things might they be doing or saying?*

*PP Um, just when I ask for things they'll be getting me things, sometimes coming up to me asking how my day is and how am I getting on and stuff like that.*

*IV And how does that feel when they come up to you…?*

*PP It feels that you're, you're important, yeah, and you've got a reason for being in here.* (Inpatient ward service user).

These staff were seen as better able to respond to individual circumstances and needs because they knew and understood people. Conversely, service users typically felt unable to form positive therapeutic relationships with staff who seemed unengaged and uninterested – that is, those who were unresponsive, didn't listen, didn't make time for people, didn't engage in small talk, and didn't get to know people. Whilst service users in crisis houses occasionally described staff as aloof or standoffish, inpatient ward service users at times described being actively – rather than passively - ignored by staff, leading to frustration and anger which sometimes spiralled into violence. Again, this mirrored staff accounts of witnessing poor practice by colleagues.

### Staff as a collective

#### Staff morale

Staff described the importance of having a good team employing a coherent and uniform approach for their ability to contain difficult situations and to build staff morale. This enhanced morale in turn had a positive impact on relationships with service users.

*Because we have a team approach, um, and, and because we work so closely together, um, hopefully our residents feel held by the expectation that things can get better and that we believe in them as, as individuals.*

(Crisis house staff member).

Staff described needing support from management and colleagues to meet the demands of the job, maintain motivation, and communicate effectively with service users.

*It's a very demanding job and I think that if you don't have good support and feel, you know, you have to have stamina in this job, I think, and if you don't feel supported, um, it can definitely have an impact on, you know, what you're willing to give, how much patience you might have for people in their presentations.*

(Inpatient ward staff member).

### Organisational functions and features

#### Deprivation of freedom

Levels of freedom granted to service users varied between crisis houses and inpatient wards, and service users reported that this could affect their opinions of staff and the capacity and potential for therapeutic alliances to form. A key difference between settings is that crisis houses do not accept compulsory admissions and do allow self-referrals, and some crisis house staff described service users' consent to treatment as important for the development of therapeutic relationships based on mutuality and consent.

*I think people probably feel a bit happier over the fact that they've got that freedom and maybe it avoids some tensions.*

(Crisis house staff member).

Crisis house service users sometimes negotiated their freedoms on a daily basis, and typically felt safe and supported as a consequence. Conversely, almost all of the acute ward service users had significant restrictions placed on their freedom, regardless of whether they were detained compulsorily. For example, voluntary service users were only able to leave the ward if a staff member was available to open the door, and access was restricted to certain areas of the ward, notably the kitchen. Most service user participants felt that this immediately established a negative dynamic between staff and service users. Some inpatient ward service users employed prison analogies, with staff likened to wardens, impeding therapeutic alliances. Others felt that the deprivation of their freedom did not have a rationale and actively undermined their chances of recovery. Many service users felt that therapeutic alliances would be enhanced by lessening restrictions on liberty, primarily because anger, frustration and aggression would be reduced, leading to a less hostile and volatile environment and more stable relationships.

*I think the ward atmosphere can feel a bit like being inside a pressure cooker, and, um, if you don't have the freedom to get out, it can lead to explosions.*

(Inpatient ward service user).

A number of inpatient ward service users described gaining leave as being *like playing chess*, undermining open and honest therapeutic alliances.

*It makes you feel like you have to convey a certain impression to them in order to win your freedom or whatever. So sometimes I think you feel like you have to engage with them in a certain way or you often hear patients here say, oh, you've got to play the game. To get out you have to play the game.*

(Inpatient ward service user).

#### Attitudes to autonomy and responsibility

Related to differing levels of freedom restrictions, levels of autonomy and responsibility experienced by service users varied between crisis houses and hospital wards. In crisis houses, service users felt that staff expected them to take personal responsibility, such as identifying post-crisis support. Whilst some welcomed this, for a minority their current difficulties meant that this expectation seemed unrealistic. On inpatient wards, service users often described a complete lack of personal autonomy. Their dependence on staff – down to the smallest things such as having to ask for a cup to make tea – was often experienced as infantilising, impeding their relationships with staff.

*It puts you in quite a… like a subordinate position when you have to knock and knock and wait for someone to look up from what they're doing, and sometimes they don't look up*

(Inpatient ward service user).

Staff in both settings felt that the severity and type of crisis being experienced by the service user impacted on the extent to which autonomy could be promoted. They felt that whilst removing responsibility could be helpful to service users when in crisis, it could also impact negatively on therapeutic alliances. Consequently, staff tried to achieve a balance between promoting autonomy and ensuring safety.

*Well no-one likes … being preached to, do they? … that kind of approach, um, forges an atmosphere of … almost, resentment, and increases the … patient/expert dichotomy. And, I know in certain cases that can be very useful, and when people are at very low ebb and they, they need …. that feeling that someone's taken the responsibility of them for a period, just for that short period*

(Crisis house staff member).

#### Staff visibility and availability

Service users in both settings felt that staff were generally available if they had an immediate need for help. However, staff were also said to spend most of their time in the office. This had a less pronounced impact in crisis houses because service users and staff reported sharing dedicated one to one time, meals and, on occasion, activities. However, on inpatient wards, a lack of staff visibility appeared to have a profound impact on service users, the ward atmosphere and therapeutic alliances.

*You can't build a relationship if you're always in the office. And, like I say, I can't really build a relationship if I'm always in my room.*

(Inpatient ward service user).

Similarly, staff in both settings highlighted the importance of spending time with service users in order to build strong therapeutic alliances.

*I think the more time you spend with a service user then the more you have a stronger bond, the more you kind of I guess understand the service user and there is kind of a stronger therapy building relationship.*

(Crisis house staff member).

Whilst crisis house staff often felt that they had this time, inpatient ward staff reported that heavy workloads, bureaucracy and limited staff numbers hindered the development of therapeutic alliances. Some staff also felt that local changes in funding and spending cuts had reduced the time they could spend with service users.

#### Contrasts in atmosphere and environment

Service users in crisis houses typically described the atmosphere as homely, relaxed and peaceful and often perceived the space as being shared with staff. Many service users felt that these factors had a positive impact on their relationships with staff. Whilst half of the service users on inpatient wards were positive about the atmosphere - describing it as relaxed, quiet, easy-going and friendly - the other half described the environment as claustrophobic and the atmosphere as volatile with constant and intrusive noise.

*The hospital, it feels, it makes, it makes you feel like you're inside of a bottle of, uh, medicine.* (Inpatient ward service user).

Staff reports similarly highlighted a distinction between the calm atmosphere of crisis houses – which were felt to impact positively on service users and therapeutic relationships - and the hectic atmosphere of inpatient wards which were seen as less relaxed and more claustrophobic. This impacted on relationships where staff had less time to talk to service users and attend to their needs

*It feels safer, I think people feeling that they can relax more.*

(Crisis house staff member).

## Discussion

### Main findings

#### The relationship between therapeutic alliance and satisfaction in inpatient wards and residential crisis alternatives

Previous studies have found higher satisfaction amongst crisis house service users than those on acute wards [12], [17], but have not produced quantitative evidence for potential explanatory mechanisms. We have generated such evidence. Firstly, better therapeutic alliances were strongly associated with greater service user satisfaction. This mirrors research findings in mental and physical healthcare [27], [28]. Secondly, in a model containing therapeutic alliance, informal peer support and staff-related negative events, the difference in satisfaction ratings between crisis houses and inpatient wards was no longer statistically significant. Clinical and demographic factors and service users' views of their recovery had little impact on satisfaction ratings in each setting. This suggests that therapeutic alliance, the quality of informal peer relationships and exposure to staff-related negative events may be important determinants of service user satisfaction with residential crisis care. Moreover, it is more fruitful to seek explanations for variance in service user satisfaction with residential inpatient care in service users' experiences, rather than in their individual characteristics.

#### Individual level factors

The qualitative phase of this study identified a number of factors which may help explain the differences in ratings of therapeutic alliance and satisfaction between crisis houses and inpatient wards arose. One of the most important determinants of therapeutic alliance was the basic personal qualities and interpersonal skills of staff: detailed accounts consistently underscored the importance of kindness, warmth, interest and engagement, and the damage caused by disinterest and disrespect. This is a commonly repeated finding in the mental health literature [6], [7], [16]. Being ignored was the most frequent negative staff-related event identified in the quantitative phase. Similarly, both service users and staff on inpatient wards described instances of service users being ignored by staff leading to frustration and anger, whilst crisis house service users sometimes found staff aloof [16].

A further important determinant of therapeutic alliance was the extent to which service users experienced staff as dedicated professionals, able to observe professional boundaries but also to step outside of these with compassion when needed. This is in keeping with calls for compassion to take centre stage in mental health care and with recent UK campaigns to achieve a focus on this [29], [30]. In contrast to this, staff – particularly on inpatient wards – were sometimes seen by service users and other staff members as being there simply to collect a wage, rather than because they were dedicated to the role. This undermined therapeutic alliances.

In keeping with the prominence of these individual level factors, service users' experiences of therapeutic alliances appeared to vary considerably by staff member. Thus whilst therapeutic alliances were generally stronger in crisis houses, strong relationships with certain staff members were formed by service users on inpatient wards.

#### Service level factors

We also found evidence that service level factors were influential determinants of therapeutic alliance. An important service level determinant of therapeutic alliance was the loss of liberty and autonomy that occurred upon entering the acute ward, whether the person was admitted through compulsion or not. The majority of service users felt that this immediately established a negative dynamic between themselves and staff which could lead to anger, frustration and aggression. This suggests that the loss of liberty experienced by mental health ward service users fundamentally disrupts the possibilities of ordinary, everyday interactions. Many service users felt that an increase in freedom and a personal sense of autonomy could greatly improve relationships between staff and service users, creating less volatile, more stable environments. Whilst staff sometimes gave similar accounts of the effects of the lack of liberty and autonomy, these were less prominent or frequent than the accounts given by service users. This suggests that the extent to which therapeutic relationships are impeded by lack of freedoms may not be fully understood by some inpatient staff. In crisis houses, service users sometimes negotiated the freedom to leave the unit on a daily basis and enjoyed greater levels of personal autonomy and a calmer, more homely environment, and this was felt to benefit therapeutic alliance. It is important to note that crisis houses are able to exercise choice over admissions, and rarely accept those who are admitted compulsorily. This suggests that inpatient ward staff may face greater difficulties in establishing therapeutic alliances. However, in the multivariable model of service user satisfaction, we found that people who were detained on wards by compulsion were no less satisfied than those who were admitted voluntarily whilst voluntary service users in the qualitative sample reported having similar restrictions placed on their freedom to compulsory service users. Thus, restrictions on freedom have an important impact on therapeutic relationships for all inpatient ward service users.

A further determinant of therapeutic alliance was the visibility and availability of staff. Service users in both settings felt that staff responded to immediate needs for help, but also spent the majority of their time in the office and were therefore not a visible presence. Crisis house staff typically felt that they were able to spend time with service users, whilst inpatient ward staff felt that they were not, largely because their available time was restricted by workloads, staffing levels, bureaucracy and funding cuts. However, the preceding Alternatives Study found that face to face contact time between crisis house and inpatient ward staff and service users was very similar, despite the same perception that crisis house staff spent more time with service users, suggesting that it is the quality, rather than the quantity, of contact time that is paramount.

### Methodological considerations

An important limitation of this study is that the populations admitted to the two settings differ. In particular, crisis houses have a degree of choice over admissions and do not admit people under compulsion, potential service users are able to self-refer and few of those admitted have a recent history of violence. Whilst we measured and adjusted for potentially confounding differences between the populations, such as demographics, diagnoses and service use, it is likely that further potential confounders have not been measured: candidates include income and social support. Thus better therapeutic relationships in crisis houses may result not only from differences between settings, but also from a service user group who are more willing and able to engage with staff. An ideal design for the elimination of confounding would be a randomised controlled trial: however, the methodological challenges in conducting such a study in this acute setting have been found to be great [31].

Human resource indicators are another set of potentially important variables not measured in our study. Qualitative interviews identified staff burnout as an important impediment to good therapeutic relationships. We did not measure staff burnout and engagement: better staff well-being and more positive attitudes in crisis houses may contribute to stronger therapeutic relationships.

A further limitation is that the inclusion criteria differed for crisis houses and inpatient wards: service users were eligible to participate if they had been resident in the crisis house for a minimum of one week (five days for one site) and a minimum of two weeks on wards. This variation reflected shorter stays in crisis houses than wards. However, adjustment for time on the ward or in the crisis house so far on this stay indicated no association with satisfaction: thus this difference is unlikely to be responsible for differences found.

With regard to our measures, we have in our modelling treated therapeutic relationship as an explanatory variable in relation to client satisfaction as an outcome. However their strong association might also be seen as resulting from them not being conceptually distinct: service users' views of the quality of relationship with clinicians might be regarded as a facet of their satisfaction, although ratings of relationships with clinicians are not incorporated in the global measure of satisfaction that we used. Whatever the precise nature of the pathway, our data strongly suggest that a service in which there are strong alliances between staff and service users is more likely to be one with which service users are satisfied, regardless of their clinical and demographic characteristics.

There are a number of strengths and limitations to our sampling strategy. For the quantitative component of the study we only interviewed those who met the inclusion criteria and agreed to participate in the study. This means that the sample is not fully representative of those using the service at the time of our study. However, the response rate was good among those meeting the inclusion criteria, we were able to recruit sufficient numbers to test our hypotheses, and there was very little missing data. For the qualitative component, staff acted as gatekeepers and this may have introduced selection bias, with staff identifying participants who they believed would give favourable reports, even though we encouraged them to approach all eligible participants. Purposive sampling however meant that we were able to recruit service users who were similar in demographic characteristics and service histories to those who had used the services in the preceding year.

Our study included a high degree of service user involvement within a multidisciplinary research team that consisted of service user researchers, clinical researchers and qualitative experts. Experienced service user researchers were involved in study design, the lead author of this paper is a service user researcher, and the qualitative interview guide derived partly from themes identified as important to service users in a preceding service user-led study [16]. We were particularly able to harness our multiplicity of standpoint and perspective when analysing and interpreting the qualitative dataset; this is a form of multiple coding which enhanced the validity and relevance of our findings [32]. Furthermore, qualitative interviews were conducted by service user researchers, and there is some evidence that service user respondents may give more candid responses to peers, further increasing the validity of our findings [33].

Whilst recall bias was minimised by interviewing service users whilst resident in the service, rather than retrospectively, there is some evidence that service users give more critical accounts of their experiences when interviewed in a neutral setting following a period of reflection [34].

Two further limitations warrant reporting. First, although the study was conducted across multiple sites, all were located in inner London which is known to be demographically distinctive. Second, the measures employed in the quantitative component have established satisfactory to good psychometric properties with the exceptions of the Negative Events Schedules for Staff and Patients (NES-S and NES-P) which remain psychometrically untested.

### Implications

This is the first major study to explore differences in therapeutic alliance and satisfaction between inpatient wards and crisis houses. Our study has found that service users in crisis houses are more satisfied and enjoy better therapeutic alliances. We further found that the personal qualities of staff - such as warmth, empathy, kindness and the ability to listen to and show an interest in others - are reported to be crucial determinants of therapeutic alliance, and that inpatient ward service users too often experience a lack of compassion and humanity from ward staff [35]. Research into the drivers behind this finding is urgently required. Establishing strong working relationships is one of the four best practice principles for recovery-oriented mental health services internationally [36], and the quality of the therapeutic relationship has been found to predict outcome in mental health settings [37], [38], [39]. It may be that some inpatient nursing staff are inherently wrong for the job, lacking the basic personal qualities that lie at the heart of strong therapeutic relationships. It is equally possible that staff are themselves retreating from stressful environments, and are experiencing high levels of burnout. Indeed, one of the components of burnout as generally measured is depersonalisation, the inability to treat people as fellow human beings [40]. Thus, addressing burnout may lead to an improvement in therapeutic alliance. Faulkner has observed that, “in order to be able to support people well, staff need to feel well supported themselves” [41]. Thus, future research should explore the possibility of introducing training and support for staff that focuses on the ways in which they engage and interact with service users and enables them to develop and enhance their therapeutic alliances. This could include field mentoring by senior colleagues to assess and provide feedback on how staff relate to service users.

Our findings also suggest that improving recruitment strategies may be an important means of improving therapeutic relationships by helping to ensure that staff with the right personal qualities are employed. The importance of workforce characteristics is increasingly emphasised in health system planning, both for in-patient mental health services [42] and physical health services [37]. The Francis Report into nursing practices and standards recommends the introduction of “an aptitude test to be taken by aspirant registered nurses prior to entering into the profession to explore the candidate's attitude towards caring, compassion and other necessary professional values”. This is consistent with systematic review evidence that experiencing connection with others and the promotion of hope and empowerment are key recovery processes [43]. It is notable that the crisis houses in our sample employed staff from a wide range of backgrounds, including those with no formal mental health qualifications. Thus, a recruitment strategy which elevates the role of personal qualities and experiences may be advantageous to therapeutic alliances and service user satisfaction. However, it is important that any changes in the hospital workforce is aimed at achieving a more appropriately skilled - rather than simply a less skilled and qualified - workforce. Beyond recruitment strategies, a range of additional initiatives warrant further research, such as developing effective feedback mechanisms [44] and service user involvement in staff training [45].

We further found that service users can develop strong and supportive informal relationships with their peers, and that this support is both unique and significant for their satisfaction. The core principles of peer support – including mutuality, reciprocity, inclusivity and a focus on recovery [45] - can mean that a different therapeutic relationship is enacted from the traditional staff-service user relationship. Repper and colleagues have identified three broad categories of peer support: informal or naturally occurring support, peer-led support programmes that run alongside statutory services, and peer support worker roles [46]. Whilst there is an increasing body of evidence regarding peer support workers, there is currently little research assessing how mental health services can best encourage and facilitate informal peer support. Thus, developing methods to foster informal peer support, and – crucially - ensuring that this is led by service users [47], could be an important means of increasing service user satisfaction with crisis care.

Future research should explore whether changes to the ward environment can enable the development of better therapeutic alliances. For instance, it may be that the calmer, more domestic atmosphere of crisis houses contributes to positive therapeutic alliances, whilst the volatile, claustrophobic atmosphere of wards undermines such relationships [20]. Furthermore, our findings confirm studies which have identified service users' loss of power, control and liberty as damaging to therapeutic alliances [8]. We further found that people who were admitted to inpatient wards voluntarily often had similar experiences to those who were detained compulsorily. This warrants investigation.

Overall, our findings join a research-derived evidence base for crisis houses, generating some confidence in crisis house models and providing an emerging explanation for why they are typically favoured by service users. Expanding the provision of local residential crisis house provision would provide greater choice to service users regarding the care they receive when in a crisis, potentially leading to a local acute care system that is better able to respond to individual needs.

## Supporting Information

Table S1 **Linear regression analyses to identify variables associated with therapeutic alliance measured by the STAR-P).**
(DOCX)

Table S2 **Linear regression analyses to identify variables associated with service user satisfaction measured by the Client Satisfaction Questionnaire (CSQ-8).**
(DOCX)

Table S3 **Linear regression analyses to identify variables associated with recovery measured by the Recovery Assessment Scale.**
(DOCX)

Table S4 **Linear regression analyses to identify predictors variables associated with informal peer support measured by the Interpersonal Relationship Inventory.**
(DOCX)
