# Correction: The Relationship between Therapeutic Alliance and Service User Satisfaction in Mental Health Inpatient Wards and Crisis House Alternatives: A Cross-Sectional Study

**DOI:** 10.1371/journal.pone.0110097

**Published:** 2014-10-01

**Authors:** The PLOS ONE Staff

The following information is missing from the Funding section: This project was funded by the National Institute for Health Research Health Services and Delivery Research Programme (project number 09/144/50).
