# Supplementary material for: The Relationship between Therapeutic Alliance and Service User Satisfaction in Mental Health Inpatient Wards and Crisis House Alternatives: A Cross-Sectional Study
Source: PLoS One. 2014 Jul 10;9(7):e100153. doi: 10.1371/journal.pone.0100153 (PMC4091866; doi:10.1371/journal.pone.0100153)
Supplement: Table S1 — Linear regression analyses to identify variables associated with therapeutic alliance measured by the STAR-P). (DOCX) [file pone.0100153.s001.docx]

**Table S1 Linear regression analysis to identify predictors of therapeutic alliance measured by the STAR-P**

| **Characteristic** | | **Coefficient (95% CI)** | **P-value** |
| --- | --- | --- | --- |
| **Service type** | ward versus crisis house | -8.74 (-12.30, -5.19) | <0.0001 |
| **Gender** | female versus male | -1.73 (-3.74, 0.29) | 0.09 |
| **Age** | per 5 years older | 0.40 (0.05, 0.15) | 0.03 |
| **Ethnic group** | White British | Ref | 0.23 |
|  | White Other | -1.91 (-5.54, 1.72) |  |
|  | Black | 0.73 (-1.90, 3.36) |  |
|  | Asian | -1.98 (-4.91, 0.94) |  |
|  | Mixed heritage | 0.54 (-3.65, 4.74) |  |
|  | Other | -1.70 (-4.68, 1.28) |  |
| **Time in service centre prior to the interview** | Per week in ward or crisis house | 0.03 (-0.09, 0.15) | 0.63 |
| **Admitted to psychiatric hospital in the past** | yes versus no | -1.26 (-3.70, 1.17) | 0.29 |
| **Mental Health Act status at admission** | detained versus not detained | 0.24 (-2.30, 2.79) | 0.84 |
| **Current/most recent clinical diagnosis** | Schizophrenia/schizo-affective | Ref | 0.73 |
|  | Bi-polar disorder | -0.49 (-3.80, 2.81) |  |
|  | Other psychosis | 2.63 (-5.72, 10.98) |  |
|  | Depression | 1.45 (-1.19, 4.10) |  |
|  | Personality disorder | 0.02 (-3.09, 3.12) |  |
|  | Other | 0.54 (-4.43, 5.50) |  |
