# Supplementary material for: The Relationship between Therapeutic Alliance and Service User Satisfaction in Mental Health Inpatient Wards and Crisis House Alternatives: A Cross-Sectional Study
Source: PLoS One. 2014 Jul 10;9(7):e100153. doi: 10.1371/journal.pone.0100153 (PMC4091866; doi:10.1371/journal.pone.0100153)
Supplement: Table S2 — Linear regression analyses to identify variables associated with service user satisfaction measured by the Client Satisfaction Questionnaire (CSQ-8). (DOCX) [file pone.0100153.s002.docx]

**Table S2: Linear regression analysis to identify variables associated with service user satisfaction measured by Client Satisfaction Questionnaire (CSQ-8)**

| **Characteristic** | | **Coefficient (95% CI)** | **P-value** |
| --- | --- | --- | --- |
| **Service type** | ward versus crisis house | -5.26 (-7.59, -2.94) | <0.0001 |
| **Gender** | female versus male | -0.81 (-2.12, 0.51) | 0.22 |
| **Age** | per 5 years older | 0.13 (-0.10, 0.35) | 0.26 |
| **Ethnic group** | White British | Reference category | 0.50 |
|  | White Other | -0.91 (-2.85, 1.03) |  |
|  | Black | -0.82 (-2.85, 1.21) |  |
|  | Asian | -1.41 (-3.55, 0.73) |  |
|  | Mixed heritage | -0.39 (-1.93, 1.16) |  |
|  | Other | -3.02 (-6.28, 0.24) |  |
| **Time in service centre prior to the interview** | per week in ward or crisis house | 0.00 (-0.08, 0.08) | 0.99 |
| **Admitted to psychiatric hospital in the past** | yes versus no | -0.67 (-2.26, 0.93) | 0.39 |
| **Mental Health Act status at admission** | detained versus not detained | -1.45 (-3.25, 0.35) | 0.11 |
| **Current/most recent clinical diagnosis** | Schizophrenia/schizo-affective | Reference category | 0.06 |
|  | Bipolar disorder | -1.91 (-3.63, -0.19) |  |
|  | Other psychosis | 3.65 (-1.02, 8.32) |  |
|  | Depression | -0.66 (-2.68, 1.36) |  |
|  | Personality disorder | -1.75 (-4.00, 0.49) |  |
|  | Other | -1.06 (-3.15, 1.03) |  |
