# Supplementary material for: The Relationship between Therapeutic Alliance and Service User Satisfaction in Mental Health Inpatient Wards and Crisis House Alternatives: A Cross-Sectional Study
Source: PLoS One. 2014 Jul 10;9(7):e100153. doi: 10.1371/journal.pone.0100153 (PMC4091866; doi:10.1371/journal.pone.0100153)
Supplement: Table S3 — Linear regression analyses to identify variables associated with recovery measured by the Recovery Assessment Scale. (DOCX) [file pone.0100153.s003.docx]

**Table S3: Linear regression analysis to identify variables associated with recovery measured by the Recovery Assessment Scale (RAS)**

| **Characteristic** | | **Coefficient (95% CI)** | **P-value** |
| --- | --- | --- | --- |
| **Service type** | ward versus crisis house | 2.36 (-4.90, 9.62) | 0.51 |
| **Gender** | female versus male | 0.39 (-8.11, 8.89) | 0.93 |
| **Age** | per 5 years older | 0.78 (-0.26, 1.81) | 0.13 |
| **Ethnic group** | White British | Reference category | 0.09 |
|  | White Other | 6.28 (-5.69, 18.25) |  |
|  | Black | 11.21 (2.66, 19.76) |  |
|  | Asian | 1.28 (-9.35, 11.91) |  |
|  | Mixed heritage | -0.49 (-11.69, 10.72) |  |
|  | Other | -0.66 (-9.55, 8.24) |  |
| **Time in service centre prior to the interview** | per week in the ward or crisis house | -0.07 (-0.44, 0.29) | 0.69 |
| **Admitted to psychiatric hospital in the past** | yes versus no | -2.02 (-9.99, 5.96) | 0.60 |
| **Mental Health Act status at admission** | detained versus not detained | 7.46 (0.71, 14.21) | 0.03 |
| **Current/most recent clinical diagnosis** | Schizophrenia/schizo-affective | Reference category | <0.0001 |
|  | Bipolar disorder | -1.29 (-11.54, 8.95) |  |
|  | Other psychosis | 3.11 (-18.10, 24.33) |  |
|  | Depression | -20.72 (-31.91, -9.53) |  |
|  | Personality disorder | -31.41 (-41.36, -21.47) |  |
|  | Other | -12.68 (-23.04, -2.31) |  |
