# Supplementary material for: The Relationship between Therapeutic Alliance and Service User Satisfaction in Mental Health Inpatient Wards and Crisis House Alternatives: A Cross-Sectional Study
Source: PLoS One. 2014 Jul 10;9(7):e100153. doi: 10.1371/journal.pone.0100153 (PMC4091866; doi:10.1371/journal.pone.0100153)
Supplement: Table S4 — Linear regression analyses to identify predictors variables associated with informal peer support measured by the Interpersonal Relationship Inventory. (DOCX) [file pone.0100153.s004.docx]

**Table S4: Linear regression analysis to identify variables associated with informal peer support measured using the Interpersonal Relationship Invenstory (IPR)**

| **Characteristic** | | **Coefficient (95% CI)** | **P-value** |
| --- | --- | --- | --- |
| **Service type** | ward versus crisis house | -12.08 (-18.53, -5.63) | 0.001 |
| **Gender** | female versus male | -2.07 (-6.39, 2.24) | 0.33 |
| **Age** | per 5 years older | -0.15 (-1.17, 0.87) | 0.76 |
| **Ethnic group** | White British | Reference category | 0.54 |
|  | White Other | -0.89 (-7.55, 5.77) |  |
|  | Black | -0.62 (-7.60, 6.37) |  |
|  | Asian | -4.49 (-12.50, 3.52) |  |
|  | Mixed heritage | -4.31 (-12.13, 3.51) |  |
|  | Other | -4.83 (-14.11, 4.44) |  |
| **Time in service centre prior to the interview** | per week in the ward or crisis house | -0.07 (-0.29, 0.16) | 0.53 |
| **Admitted to psychiatric hospital in the past** | yes versus no | -4.23 (-7.79, -0.68) | 0.02 |
| **Mental Health Act status at admission** | detained versus not detained | -0.55 (-6.71, 5.62) | 0.86 |
| **Current/most recent clinical diagnosis** | Schizophrenia/schizo-affective | Reference category | 0.15 |
|  | Bipolar affective disorder | -2.30 (-11.10, 6.51) |  |
|  | Other psychosis | 5.61 (-3.36, 14.58) |  |
|  | Depression | -2.63 (-8.47, 3.20) |  |
|  | Personality disorder | -6.46 (-12.76, -0.17) |  |
|  | Other | -1.50 (-8.10, 5.10) |  |
